# Association between paced QRS duration and atrial fibrillation after permanent pacemaker implantation: A retrospective observational cohort study

**DOI:** 10.1097/MD.0000000000009839

**Published:** 2018-02-09

**Authors:** FuWei Xing, JingZhou Jiang, XiaoLiang Hu, Chong Feng, JianGui He, YuGang Dong, YueDong Ma, AnLi Tang

**Affiliations:** a Department of Cardiology, The First Affiliated Hospital of Sun Yat-Sen University; b Key Laboratory on Assisted Circulation, Ministry of Health; c Department of Cardiology, The Third Affiliated Hospital of Sun Yat-Sen University, Guangzhou, People's Republic of China.

**Keywords:** atrial fibrillation, atrioventricular block, electrocardiography, pacemaker, QRS duration

## Abstract

Right ventricular pacing often results in prolonged QRS duration (QRSd) as the result of right ventricular stimulation, and atrial fibrillation (AF) may result. The association of pacing-induced prolonged QRSd and AF in patients with permanent pacemakers is unknown.

We selected 180 consecutive patients who underwent pacemaker implantation for complete/advanced atrioventricular block. All of the patients were paced from the right ventricular septum. Electrocardiography recordings were obtained at the beginning and the end of pacemaker implantation. QRSd was measured in all 12 leads. The QRSd variation was calculated by subtracting the preimplantation QRSd from the postimplantation QRSd.

The occurrence of AF was observed in 64 (35.56%) patients (follow-up 33.62 ± 21.47 mo). No significant differences in preimplantation QRSd were observed between the AF occurrence and nonoccurrence groups. The QRSd variation in leads V4 (54.22 ± 29.03 vs 42.66 ± 33.79 ms, *P* = .022), and V6 (64.62 ± 23.16 vs 48.45 ± 34.40 ms, *P* = .001) differed significantly between the occurrence and nonoccurrence groups. More QRSd variation in lead V6 (*P* = .005, HR = 1.822, 95% CI 1.174–2.718, interval scale of QRSd was 40 ms) and left atrial diameter (*P* = .045, HR = 1.042, 95% CI 1.001–1.086) were independent risk factors for AF occurrence. Receiver operating characteristic curve suggested that QRSd variation in lead V6 could predict AF occurrence, especially for patients with long preimplantation QRSd (≥120 ms, area under the curve was 0.826, 95% CI 0.685–0.967).

QRSd variation in lead V6 might be positively correlated with postimplantation AF occurrence. In patients with pacemaker implantation, QRSd could be a complementary criterion for optimizing the right ventricular septal pacing site, and smallest QRSd might be worth pursuing.

## 1 Introduction

Previous trials have revealed that atrial fibrillation (AF) is a “negative effect” associated with right ventricular (RV) pacing, and there was a linearly increasing relationship between the cumulative percentage of RV pacing and risk of AF. Novel pacing modes which aim to minimize unnecessary RV pacing have been developed. However, RV pacing cannot be avoided or minimized in patients with permanent complete heart block.^[1]^

One recent study has suggested that alternative RV pacing sites might be associated with the risk of AF. Hisian area pacing compared with RV apex or RV septal pacing seems to be associated with a lower risk of AF occurrence.^[2]^ However, Hisian area pacing may be technically difficult and may not be adequate in patients presenting with infra Hisian conduction problems.^[1]^ Considering the strong evidence of harm with apical pacing, the septum and the RV outflow tract have been suggested as alternative pacing sites.^[1]^

Is there any way to reduce the risk of AF occurrence for pacemaker (PM) patients who could neither minimize unnecessary RV pacing nor choice Hisian area pacing? We hypothesized that there is a relationship between paced QRS duration (QRSd) and postimplantation AF occurrence.

One previous study has suggested that RV lead implantation guided by surface QRSd is feasible.^[3]^ In their study, QRSd was the criterion for optimizing the RV pacing site. Mapping of the interventricular septum was performed by means of custom shaped stylets until the smallest QRSd available was recorded. A complementary electrocardiography (ECG) criterion for optimizing the RV septal pacing site might be necessary. And smaller QRSd might be associated with a lower risk of AF occurrence.

## 2 Materials and methods

### 2.1 Study population

We retrospectively analyzed 180 consecutive patients who underwent PM implantation for complete/advanced atrioventricular block at the First Affiliated Hospital of Sun Yat-sen University from January 2010 to June 2016.

The exclusion criteria were as follows: previous history of AF, implantable cardioverter defibrillator, or indication for cardiac resynchronization therapy, significant valve disease (mitral or aortic regurgitations/stenosis of grade moderate or severe), heart surgery within the last 6 months before PM implantation, absence of high percentage of ventricular pacing (≥40%) as observed at each follow-up, and poor-quality ECG.

All patients were informed of the investigation and nature of the implantation, and written informed consent for implantation was obtained. And all experimental protocols complied with institutional ethical committees for Clinical Research and Animal Trails of the First Affiliated Hospital of Sun Yat-Sen University and FDA guidelines.

### 2.2 Implantation procedure and lead placement

Double-chamber PM systems were performed by a group of operators experienced in lead placement. Prophylactic intravenous antibiotics were given half an hour before the procedure. PM implantation procedure was done under local anesthesia. The RV lead was inserted via the left- or right side subclavian venous approach.

All of patients were paced from the RV septum. Lead placement was performed using a conventional 7-French active-fixation lead in all patients. Lead positions were confirmed in the left anterior oblique and right anterior oblique fluoroscopic views during implantation. No specific ECG criteria of final lead position were given. The target RV septal pacing site was in the mid-upper third of the RV septum determined by dividing the RV septum into thirds in the left anterior oblique >30 degrees fluoroscopic projection.^[2]^ Once the tip of the RV lead made attachment with septal positioning, the screw was deployed. And posteroanterior and lateral chest x-rays were performed in all patients undergoing pacing to corroborate the pacing site.

### 2.3 Postimplantation follow-up

All the enrolled patients were followed for at least for 12 months. All patients were in sinus rhythm at the time of PM implantation. Before hospital discharge, clinical evaluation and echocardiograms were performed.

Follow-up were performed at 1 and 3 months postimplantation and every 6 months thereafter. ECG was performed at each visit. Five weeks was defined as the blanking period.^[2]^ No data regarding AF episodes was collected during blanking period after device implantation. The maximum tracking rate was individualized and the mode switch function was activated. Mode switch occurred, when the atrial rate exceeded 170 to 180 beats per minute for a given number of beats or period of time according to the settings of the manufacturer of the PM. AF occurrence was defined as any episode of mode switch at least 5 minutes in follow-up duration after the blanking period.^[4,5]^

The follow-up was also conducted to determine the maximum percentage of ventricular pacing and the percentage of atrial pacing beats. High percentage of ventricular pacing was defined as ≥40%.^[6]^

### 2.4 Electrocardiography recording and data analysis

Standard 12-lead ECG measurements were recorded at the beginning and the end of the device implantation. Then ECGs were digitized and measured using Engauge Digitizer 5.1 software (M. Mitchell, Engauge Digitizer, http://digitizer.source-forge.net).^[7]^ All ECG recordings were measured by 2 independent readers who were blinded to this clinical research. We measured QRSd in 12 leads and expressed the results in milliseconds. The preimplantation QRSd (QRSd_pre_) was measured from the earliest onset to the latest deflection of the QRS complex.^[8]^ The paced QRSd (QRSd_paced_) was measured from the beginning of the ventricular pacing spike to the end of the QRS complex.^[9]^ In each lead, the mean value for 3 consecutive complexes was defined as the final QRSd. Then we computed the average values (QRSd_mean_) of 12 leads. The QRSd_max_ was measured from the earliest onset in any lead to the latest deflection in any lead.^[8]^ The QRSd variation was measured by subtracting the QRSd_pre_ from the QRSd_paced_ (QRSd_paced_ – QRSd_pre_).

### 2.5 Statistical analysis

Statistical analysis was performed using SPSS 12.0 (SPSS Inc., Chicago, IL) and Stata 12 (StataCorp, College Station, TX). Continuous variables were presented as means ± standard deviation. Abnormally distributed data were expressed as medians (upper and lower quartiles). Between-group comparisons of normally distributed data were performed using independent-sample *t* tests. The Mann-Whitney test was used to analyze abnormally distributed data. And the comparison among groups of enumeration data was tested by chi-square. Pearson correlation coefficient was used to test the homogeneity of QRSd data between 2 independent readers.

Cox's proportional hazard model was used to estimate hazard ratio of occurrence of AF for QRSd variation in lead V6 adjusted for various potential confounders selected by forward stepwise regression method between: QRSd variation and QRSd_paced_ in lead V4, QRSd_paced_ in lead V6, percentage of atrial and RV pacing, left atrial diameter, left ventricular ejection fraction, age, QRSd_mean-pre_ (the average value of the 12 leads preimplantation QRSd, was assessed as a dichotomous variable, ≥120 ms or <120 ms), left ventricular end-diastolic diameter, diabetes, hypertension, coronary artery disease, use of angiotensin I-converting enzyme inhibitory or angiotensin II receptor blocker, use of beta-blockers, and use of calcium antagonists. The Cox proportional hazards model depends on the assumption of a constant hazard over time, which was evaluated by a global goodness of fit test proposed by Schoenfeld.^[10,11]^

A receiver operating characteristic (ROC) curve was constructed to evaluate the sensitivity and specificity of various cut-off values of QRSd indices for predicting postimplantation AF occurrence. Statistical significance was denoted at *P* < .05.

## 3 Results

### 3.1 Procedural characteristics and atrial fibrillation occurrence

A total of 185 adult consecutive patients underwent permanent RV septal pacing for complete/advanced atrioventricular block (between January 2010 and June 2016). Five patients were excluded from our study for dislodgement of the RV lead, which was managed by replacing the lead close to the original site (RV septum).

The study cohort was composed of 180 patients. Seven patients were infected after PM implantation and all of them received antibiotics. Among them, 1 patient became chronically infected. During a mean follow-up of 33.62 + 21.47 months, AF occurred in 64 patients (35.56%). Among patients with AF occurrence postimplantation, 22 were prescribed anticoagulant (warfarin, dabigatran, or rivaroxaban) according to CHADS_2_ value.^[12]^ The clinical characteristics of all patients, and the AF occurrence and nonoccurrence groups are presented in Table 1. Age, sex, left atrial diameter, and body mass index were not significantly associated with AF occurrence, whereas follow-up time was longer in AF occurrence group compared with nonoccurrence group (*P* = .009).

**Table 1:**
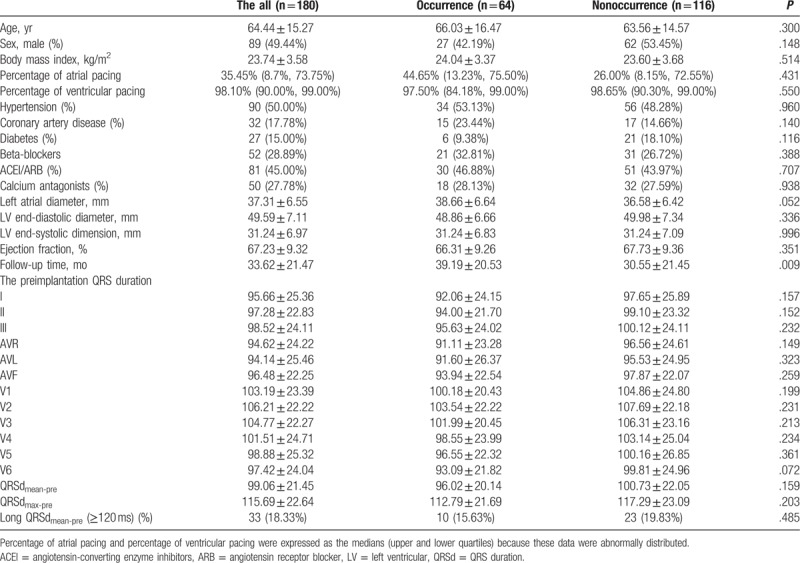
Baseline characteristics and preimplantation QRS duration.

### 3.2 QRS duration before and after implantation

The Pearson correlation coefficient for QRSd data between 2 independent readers was 0.94 (*P* < .001). A comparison of QRSd data between the occurrence and nonoccurrence groups indicated that QRSd_pre_ did not differed in any of the 12 leads between the 2 groups (Table 1), whereas QRSd_paced_ differed significantly in leads V4 (152.77 ± 17.04 vs 145.80 ± 23.16 ms, *P* = .036), and V6 (157.71 ± 14.99 vs 148.26 ± 23.62 ms, *P* = .004) between the 2 groups (Table 2). The QRSd variation in leads V4 (54.22 ± 29.03 vs 42.66 ± 33.79 ms, *P* = .022), and V6 (64.62 ± 23.16 vs –48.45 ± 34.40 ms, *P* = .001) differed significantly between the 2 groups (Table 2). A tendency toward longer QRSd variation was observed in all other leads but did not reach statistical significance (Table 2). Cox univariate analysis suggested the QRSd_paced_ and QRSd variation in leads V6 differed significantly between the 2 groups (*P* = .029 and .002, respectively) (Table 2).

**Table 2:**
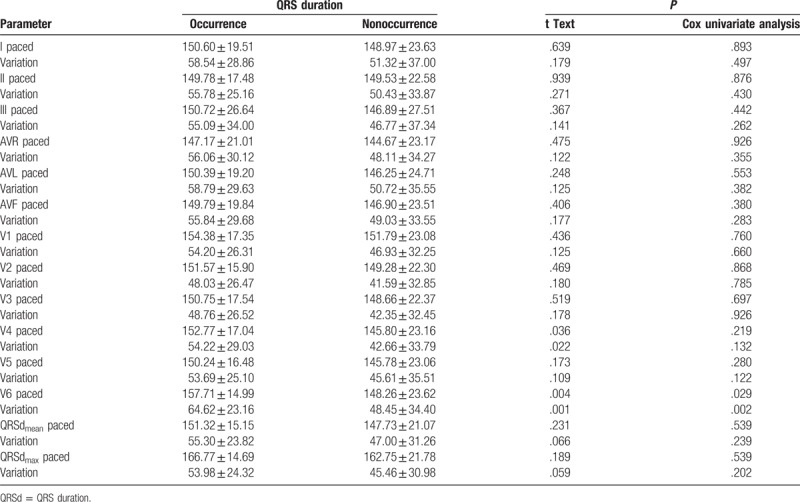
Comparison of paced QRS duration and QRS duration variation between occurrence and nonoccurrence group.

Consequently, these 4 parameters (QRSd_paced_ in leads V4 and V6, QRSd variation in leads V4 and V6) were introduced in the Cox proportional hazard model (Table 3). Moreover, percentage of RV pacing, percentage of atrial pacing, left atrial diameter, left ventricular ejection fraction, QRSd_mean-pre_, and age were introduced in Cox model. And it concluded that a longer QRSd variation in lead V6 (*P* = .005, HR = 1.015, 95% CI 1.004–1.025) and left atrial diameter (*P* = .045, HR = 1.042, 95% CI 1.001–1.086) independently predicted postimplantation AF occurrence. In order to approve the proportional hazards assumption, a global goodness of fit test proposed by Schoenfeld was done.^[10,11]^ According to this test, proportionality assumption was confirmed for all covariates and whole model (*P* = .990).

**Table 3:**
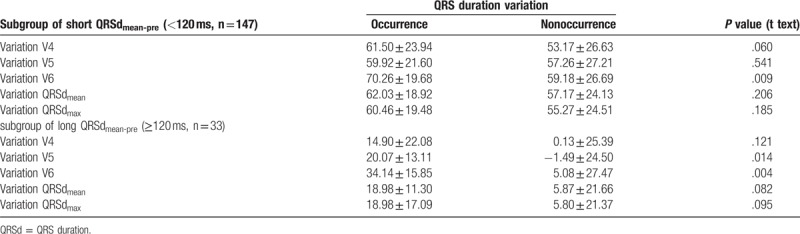
Comparison of QRS duration variation between occurrence and nonoccurrence group in subgroup analysis.

Hazard ratio was calculated via following calculation formula:
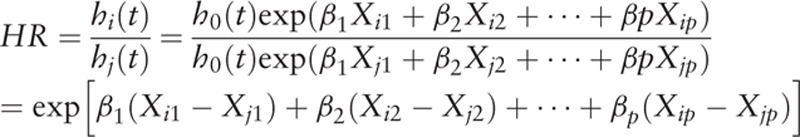


Among this calculation formula, exp(*β*_*j*_) represented the value of hazard ratio which caused by every 1 unit increment of variable quantity *X*_*j*_, when other covariant quantities remained unchanged. So HR of QRSd variation in lead V6 was caused by every increase of 1 number in millisecond. According to medical knowledge, interval scale of QRSd was defined as 40 ms (*k* = 40). Then we calculated HR’ via following calculation formula:
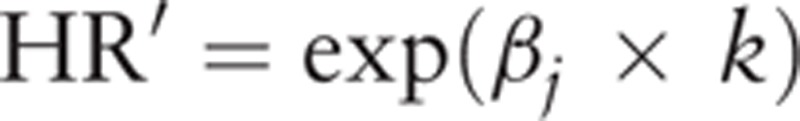


HR’ of QRSd variation in lead V6 was 1.814, and 95% confidence interval was 1.221 and 2.685, respectively.

### 3.3 Subgroup analysis (QRSd_mean-pre_ ≥ or <120 ms)

All of patients were divided into 2 subgroups according to long QRSd_mean-pre_ (≥120 ms, n = 31) and short QRSd_mean-pre_ (<120 ms, n = 149). Independent-sample t tests and Cox model were performed again (Tables 3 and 4).

**Table 4:**
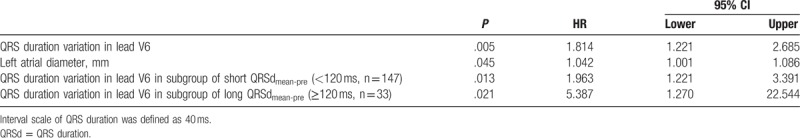
Cox proportional hazard model: predictors of postimplantation atrial fibrillation occurrence.

In subgroup of short QRSd_mean-pre_ (<120 ms), the QRSd variation in leads V6 (70.26 ± 19.68 vs 59.18 ± 26.69 ms, *P* = .009) differed significantly between the 2 groups. And Cox model concluded that a longer QRSd variation in lead V6 (*P* = .013, HR = 1.017, 95% CI 1.004–1.031) independently predicted AF occurrence. When interval scale of QRSd was defined as 40 ms, HR’ was 1.963 (95% CI 1.221–3.391).

In subgroup of long QRSd_mean-pre_ (≥120 ms), the QRSd variation in leads V5 (20.07 ± 13.11 vs −1.49 ± 24.50 ms, *P* = .014), and V6 (34.14 ± 15.85 vs 5.08 ± 27.47 ms, *P* = .004) differed significantly between the 2 groups. And Cox model concluded that a longer QRSd variation in lead V6 (*P* = .021, HR = 1.043, 95% CI 1.006–1.081) independently predicted AF occurrence. When interval scale of QRSd was defined as 40 ms, HR’ was 5.387 (95% CI 1.270–22.544).

### 3.4 Value of QRS duration parameter for predicting postimplantation atrial fibrillation

**Figure 1:**
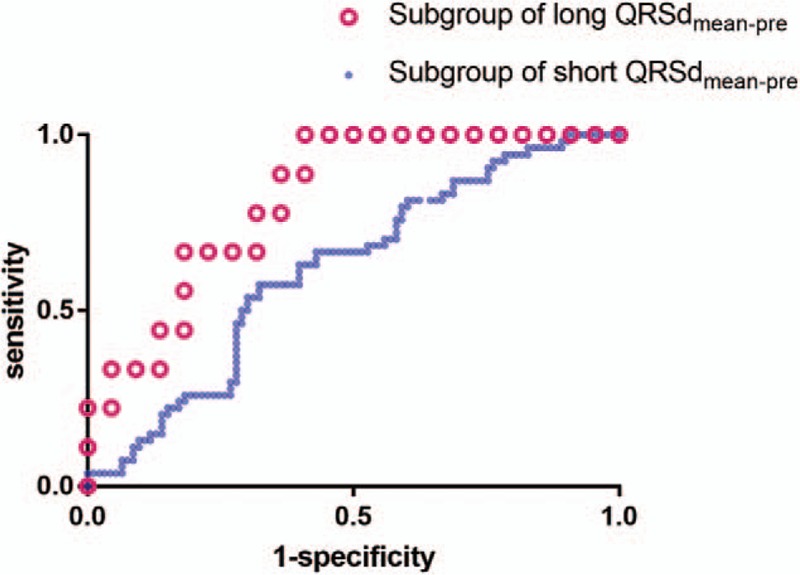
Receiver operating characteristic curves for relationship between QRS duration variation in leads V6 and atrial fibrillation occurrence. All of patients were divided into 2 subgroups according to long QRSd_mean-pre_ (≥120 ms, the area under the curve was 0.826) and short QRSd_mean-pre_ (<120 ms, the area under the curve was 0.616). QRSd = QRS duration.

ROC curve analysis was performed to evaluate the ability of the QRSd variation in leads V6 to predict AF occurrence (Fig. 1). In subgroup of short QRSd_mean-pre_ (<120 ms), the area under the curve for the QRSd variation was 0.616 (95% CI 0.525–0.708). A QRSd variation ≥68.2 ms in lead V6 exhibited the best combined sensitivity and specificity for AF occurrence (57.4% and 67.7%, respectively). In subgroup of long QRSd_mean-pre_ (≥120 ms), the area under the curve was 0.826 (95% CI 0.685–0.967). A QRSd variation ≥11.8 ms exhibited the best combined sensitivity and specificity for AF occurrence (100% and 40.9%, respectively).

## 4 Discussion

Previous studies have demonstrated that the cumulative percentage of RV pacing and alternative RV pacing sites might be related to the risk of AF occurrence.^[2,13]^ And QRSd was correlated with AF occurrence in patients with heart failure.^[14]^ However, there is no published data on the association of AF occurrence and paced QRSd in patients with PM. Our study suggested that the QRSd variation in lead V6 was positively correlated with postimplantation AF occurrence, and QRSd could be a complementary criterion for optimizing the RV septal pacing site.

### 4.1 Right ventricular septal pacing

The RV septum is a relatively large area and fluoroscopy could not take into account the various anatomic variations of the region. RV septal pacing consist of a heterogenous group of pacing sites, ranging from the RV free wall to the midseptal segment and even in the free wall of the true outflow tract. In addition, the RV septum can be paced from high, low, and midseptal positions.^[15,16]^ And in some studies, low part of RV septum was classified as RV apical portion.^[2]^ The conflicting data regarding RV septal and RV apical pacing might be contributed to the multiplicity of possible lead positions in RV septum despite careful positioning in the fluoroscopy projection. For example, Shimony et al^[17]^ concluded that left ventricular function was worse with RV apical than with RV nonapical pacing. By contrast, Ng et al^[15]^ concluded that RV apical pacing group had better left ventricular function than RV septal pacing group. In addition, although Pastore et al concluded that the site of RV pacing might affect the risk of AF, no statistically significant difference in the risk was observed between RV apical and RV septal groups. And Pastore et al^[2]^ suggested that it might be due to a high degree of heterogeneity in RV septal pacing sites.

It is not clear that whether these different RV septal locations yield different effects to AF occurrence by variation of QRSd_paced_. This question should be discussed further in a large prospective study.

### 4.2 Clinical significance of QRS duration in pacemaker patients

Malecka et al^[18]^ suggested that there was a correlation between shortened QRS complex duration and improvement of left ventricular ejection fraction in PM patients. Sakatani et al^[19]^ suggested that shorter QRSd was associated with better prognosis in patients with PM. And our results suggested that the QRSd variation in lead V6 could predict AF occurrence in patients with PM, especially for patients with long QRSd_mean-pre_ (≥120 ms).

Given that there was no specific ECG criteria of final lead position in RV septum in previous studies.^[2,20]^ These results raise the possibility that, in the future, we might need to optimize the RV septal pacing site based on conventional x-ray and complementary ECG (QRSd_paced_) in individual patients. Schwaab et al suggested that RV lead implantation guided by surface QRSd was feasible. Mapping of the interventricular septum was performed by means of custom-shaped stylets until the smallest QRSd available was recorded.^[3]^ And our results of ROC curve suggested that during RV septal lead placement, variation of QRSd in lead V6 should be <11.8 ms in patients with long QRSd_mean-pre_ and be <68.2 ms in patients without.

### 4.3 Relationship between QRS duration and atrial fibrillation in patients with pacemaker

QRSd represents the electrical activation of both the left and right ventricles. Although the relationship between QRSd and AF in patients with heart failure has already been clearly identified.^[14]^ Long QRSd obtained by artificial stimulation is completely different with long QRSd on the patients with heart failure. In patients with PM, the pathways of left ventricular activation are different from normal. It is supposed that the more myocardium activated by muscle conduction before the ectopic activation front enters the specialized conduction system, the longer the QRSd.^[3]^

The pathogenesis of QRSd variation and its association for AF occurrence after implantation remains unclear. The underlying mechanism may involve ventricular dysfunction and dyssynchrony. First, with long period of ventricular pacing, the underlying ventricular dysfunction contributes to left atrial remodeling/stiffness further decreasing the left atrial function. Then reduced left atrial reservoir function estimated by the total left atrial emptying fraction markedly increases the propensity for first AF or atrial flutter.^[21]^ Previous studies suggested that shortened QRSd was related to homogenization of left ventricular contraction and improvement of systolic function in patients with PM.^[3,18]^ Second, QRSd was correlated with interventricular dyssynchrony in patients with PM.^[15]^ And ventricular dyssynchrony could facilitate the onset of AF.^[14]^

### 4.4 Lead V6

Our results revealed that only lead V6 QRSd was associated with postimplantation AF occurrence. First, precordial leads (V1, V2, V3, V4, V5, and V6) could record the electrical activity of the myocardial wall directly below the exploring electrode, whereas peripheral leads (I, II, III, AVR, AVL, and AVF) could not explore specific segments of the myocardium but the whole electrical activity of the heart. Second, right precordial leads (V1, V2), exploring thinner myocardial areas, has a shorter duration than that of the left precordial leads (V5, V6). For example, intrinsicoid deflection in leads V1 and V2 is <35 ms, whereas in V5 and V6 it is <45 ms.^[22]^ Third, lead V6 is the furthest precordial ECG leads from the RV septal lead placement. As a result, any QRSd change induced via PM may be magnified in lead V6.

### 4.5 Long QRS duration

Long QRSd_mean-pre_ (≥120 ms) reflects various ventricular conditions, such as conduction disturbance, ventricular fibrosis, and mechanical left ventricular dyssynchrony.^[19]^ And these conditions could facilitate the onset of AF.^[14]^ By contrast, Pastore et al^[2]^ suggested that the presence of bundle branch block was associated with a lower risk of AF in patients with PM.

However, our Cox model suggested that the presence of long QRSd_mean-pre_ (≥120 ms) had nothing to do with AF occurrence. And our subgroup analysis suggested that, with or without the presence of long QRSd_mean-pre_ (≥120 ms), a longer QRSd variation in lead V6 independently predicted postimplantation AF occurrence.

## 5 Limitations

There were several limitations in this study. This study featured a retrospective design and was conducted at a single center with highly selected patients. For subgroup of short QRSd_mean-pre_ (<120 ms), area under the curve was just only 0.616. This might be contributed to several confounding factors that would inevitably contribute to AF occurrence, such as percentage of RV pacing, percentage of atrial pacing, left atrial diameter, left ventricular ejection fraction, age, and so on.^[2,13]^ Furthermore, to improve the reliability of our conclusion, these factors were introduced in our logistic analysis. However, the difficulty in making adequate adjustment for the different populations strongly suggests that the analysis of the data should be confirmed in a large, multicenter prospective study. Moreover, because of the small sample size in subgroup of long QRSd_mean-pre_ (n = 31), greater caution should be applied to the results of this subgroup.

## 6 Conclusion

An increase in QRSd post-implantation compared to preimplantation in lead V6 might be positively correlated with postimplantation AF occurrence. In patients with PM implantation, QRSd could be a complementary criterion for optimizing the RV septal pacing site. Considering this was a retrospective study, a large, multicenter prospective cohort study might be necessary to confirm the association between QRSd variation and postimplantation AF occurrence.
